# Anti-proliferative activities of *Byrsocarpus coccineus Schum. and Thonn.* (Connaraceae) using ovarian cancer cell lines

**DOI:** 10.1186/s13048-020-00679-8

**Published:** 2020-07-21

**Authors:** Caroline E. Ukwade, Osaretin A. T. Ebuehi, Rahmat A. Adisa, Santosh K. Singh, Rajesh Singh

**Affiliations:** 1 Department of Biochemistry, College of Medicine, University of Lagos, P.M.B, Lagos, 12003 Nigeria; 2 Department of Microbiology, Biochemistry & Immunology, Cancer Health Equity Institute, Morehouse School of Medicine, 720 Westview Drive SW, Atlanta, GA 30310 USA

**Keywords:** *B. coccineus*, Ovarian cancer, Proliferation, Apoptosis, p53, Cell cycle

## Abstract

**Background:**

Ovarian cancer (OvCa) is one of the most lethal tumors of gynecologic malignancies, due to lack of early detection, and a high rate of metastasis. The standard treatment for OvCa is surgery and cytotoxic chemotherapy. However, to overcome the high cost and side effects of these treatments, medicinal plants are widely used in developing countries to treat OvCa. *Byrsocarpus coccineus* plant preparation has been administered to patients traditionally in the management of tumors in Nigeria. In this study, we investigated the anti-proliferative effects of *B. coccineus* ethanol leaf extract against OVCAR-3 and SW 626 OvCa cell lines. After the treatment of the two cell lines with the extracts, analyses were carried out to determine inhibition of proliferation and expression of cell cycle markers, pro-apoptotic, and anti-apoptotic markers.

**Results:**

Results showed that *B. coccineus* ethanol leaf extract, significantly inhibited cell migration and colony formation in OVCAR-3 and SW 626 treated cells in a dose-dependent manner. Results also show that *B. coccineus* ethanol leaf extract modulated the expression of tumor suppressor gene (p53), cell cycle progression, pro- and anti-apoptotic gene, and the pro-inflammatory cytokines.

**Conclusions:**

These results suggest that *B. coccineus* have anti-proliferative properties and could induce apoptosis. Further investigation will be carried out to isolate bioactive compounds for the treatment of ovarian cancer.

## Background

Ovarian cancer is the fifth most aggressive and lethal cancer worldwide in women [1], due to a lack of early detection and a high rate of metastasis [2]. Epithelial ovarian carcinomas make up to 90% of malignant OvCa and it is the most aggressive [3]. In 2019, 22,530 new cases and 13,980 deaths due to OvCa were estimated to occur in the United States [4]. The standard treatment for OvCa is surgery and chemotherapy [5], but relapse occurs in most women [1].

Due to poor prognosis, cost, and side effects of treatment, phytochemicals have become a growing source of alternate medicine in Africa. *B. coccineus* plant extract preparation is administered to patients traditionally in the management of cancer in Africa. *B. coccineus* known as ‘Amuje wewe or Ado kanti-kanti’ is a scandent shrub and it is indigenous to Nigeria (West Africa). Studies have shown that the plant has anti-plasmodia [6], antimicrobial [7], and anti-diarrhea activity [8]. Fractions of *B. coccineus* have been reported to modulate cytochrome P450 (CYP) enzyme activity, cytokine production, and anti-proliferation in colon cancer cell lines [9]. Studies have also shown that the plant extract has cytotoxic activity against human breast and prostate carcinoma cell lines [10]. Activation of p53 (a tumor suppressor protein) signaling pathway inhibits cancer cell proliferation by cell cycle arrest and induction of apoptosis through the intrinsic and extrinsic pathway [11]. The p53 protein is a key regulator of apoptosis and has been implicated in the development of OvCa [1].

Therefore, the study is to justify the folkloric use as an anti-tumor plant and propose a mechanism/pathway of action of the extracts by investigating p53 involvement in cell cycle arrest and induction of apoptosis [11].

## Results

### *B. coccineus* ethanol leaf extract induces cell cytotoxicity in ovarian cancer cells

To explore the therapeutic potential of *B. coccineus* ethanol leaf extract, cell viability assay was performed for OVCAR-3 and SW 626 cells. We determined the inhibitory concentration (IC_50_ value) of extract after treatment with different concentrations at three different time points (24, 48, and 72 h). Among the treatments, we found significant cytotoxicity at 48 h compared to other time points. DMSO was used as vehicle control in untreated cells. The IC_50_ values of extract were found to be 446.5 μg/mL and 486.94 μg/mL for OVCAR-3 and SW 626 cells, respectively. However, there was no significant difference in cell death noted between 48 and 72 h time point (**see** Additional file 1). These results indicate that *B. coccineus* ethanol leaf extract inhibits the proliferation of OVCAR-3 and SW 626 in a dose and time-dependent manner.

**Fig. 1:**
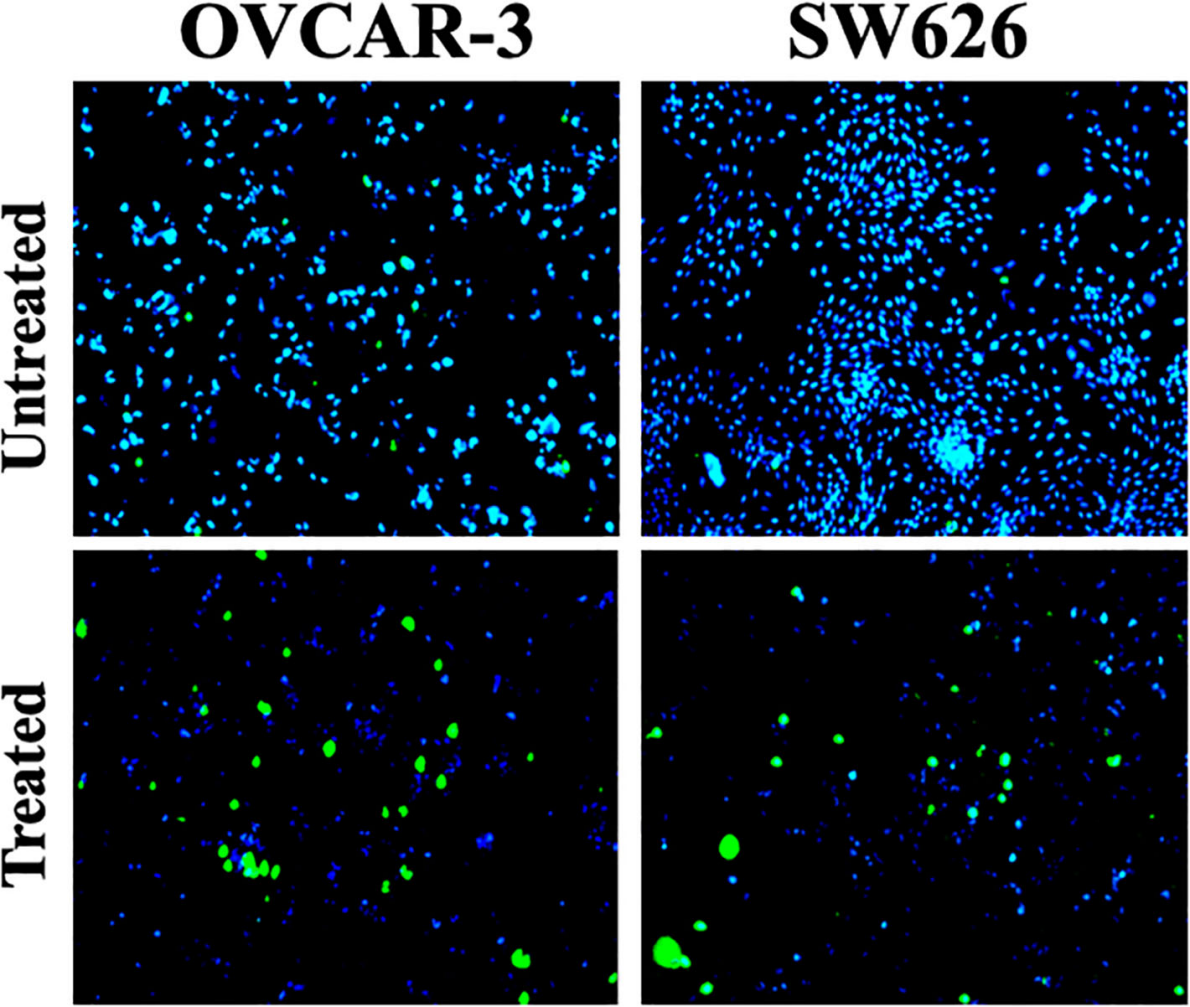
Effect of *B. coccineus* ethanol leaf extract on cell cytotoxicity in ovarian cancer cells. OvCa cells were treated with different dosage of *B. coccineus* extract (OVCAR-3: 446.5 μg/mL; and SW 626: 486.94 μg/mL) for 48 h and were processed for live/ dead cells staining. DMSO was used as vehicle control in untreated cells. Blue and green color represents live and dead cells nuclei. Immunofluorescent images showed abundant number of live nuclei in untreated cells compared to any treatment groups of OVCAR-3 and SW 626 cells. Images were captured at 4x objectives. Scale bar represents 50 μm

Considering these facts that *B. coccineus* has a cytotoxic effect on OvCa cells, we treated both cell lines with their IC_50_ values for 48 h and examined them through a cell viability staining test. As shown in Fig. 1, stained cells displayed blue and green color which represents live and dead cells nuclei, respectively. These immunofluorescent images further confirm that both cell lines have a high degree of dead nuclei when treated with their IC_50_ values compared to untreated cells. These results suggest the effectiveness of *B. coccineus* leaf extract in OvCa cells.

### *B. coccineus* leaf extract suppresses OvCa cell migration

**Fig. 2:**
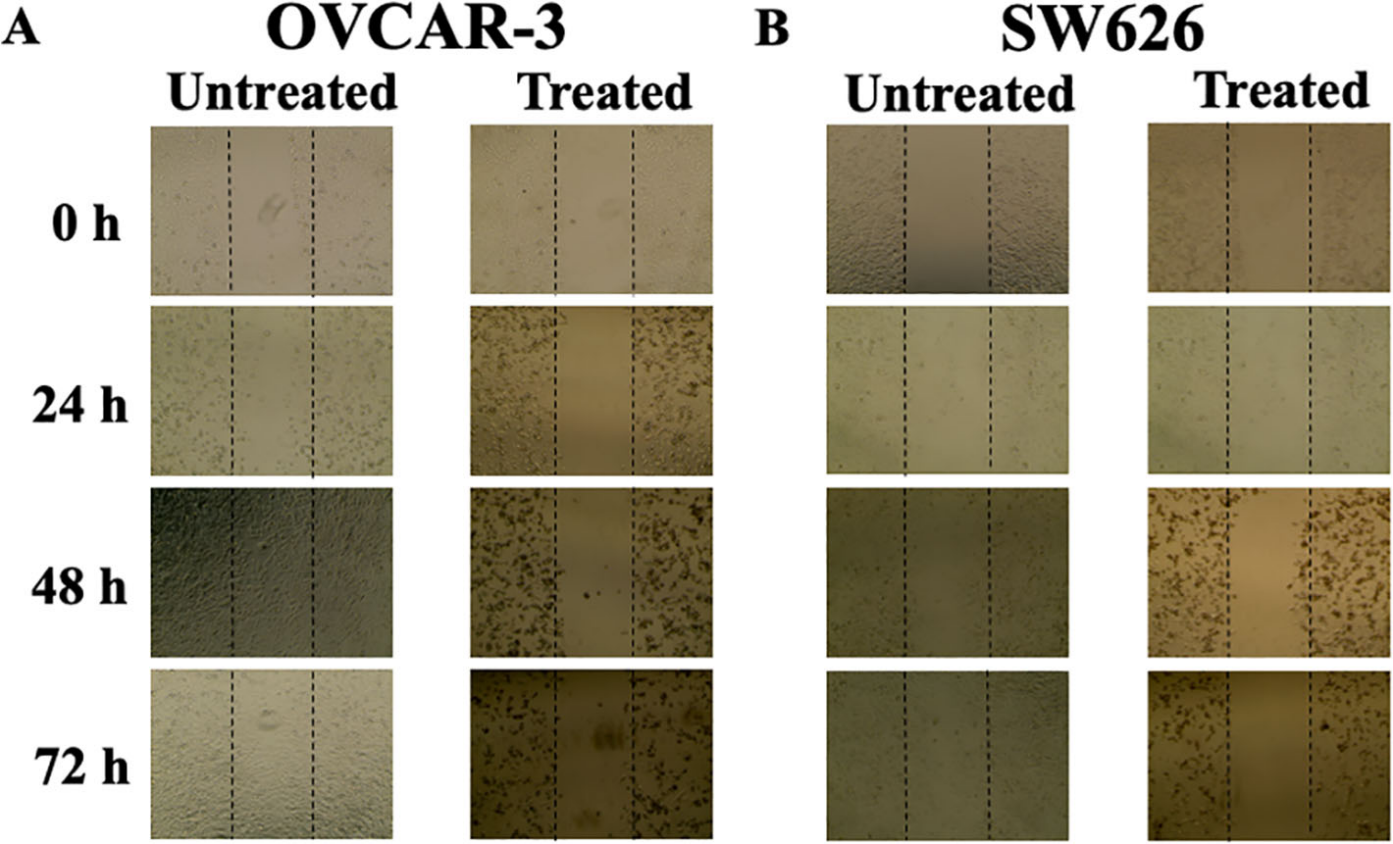
*B. coccineus* ethanol leaf extract reverses the migration of ovarian cancer cells. (**a**) OVCAR-3 (**b**) SW 626 cells monolayers were scratched using a pipette tip and then treated with *B. coccineus* extract for 0, 24 and 48 and 72 h. The representative’s images of the migratory cells showed prior to or after scratch of cells under a microscope at 10x objective. DMSO (1%) was used in untreated or control cells

*Invitro* wound healing assay is one the most carried out to measure the directional migration capacity of the cells. The assay has been reported particularly in studies on the effects of cell-matrix and cell-cell interactions on cell migration [12]. To investigate the role of *B. coccineus* ethanol leaf extract on OvCa cells migration, scratch assay was performed in OVCAR-3 and SW 626 cells. As shown in Fig. 2, extract was able to inhibit cell migration in both OvCa cells compared to untreated control in a dose and time-dependent manner. In the baseline control the average gap distance was found to be 69.25 μm. After the scratch and incubating cells for 24, 48 and 72 h, there was no significant difference in the gap distance between treated cells, while the gap was completely closed in the untreated cells when compared to zero hours. It could be inferred from the results, that *B. coccineus* ethanol leaf was able to inhibit cell migration in OVCAR-3 and SW 626.

### *B. coccineus* extract decreased colony formation and cell growth

**Fig. 3:**
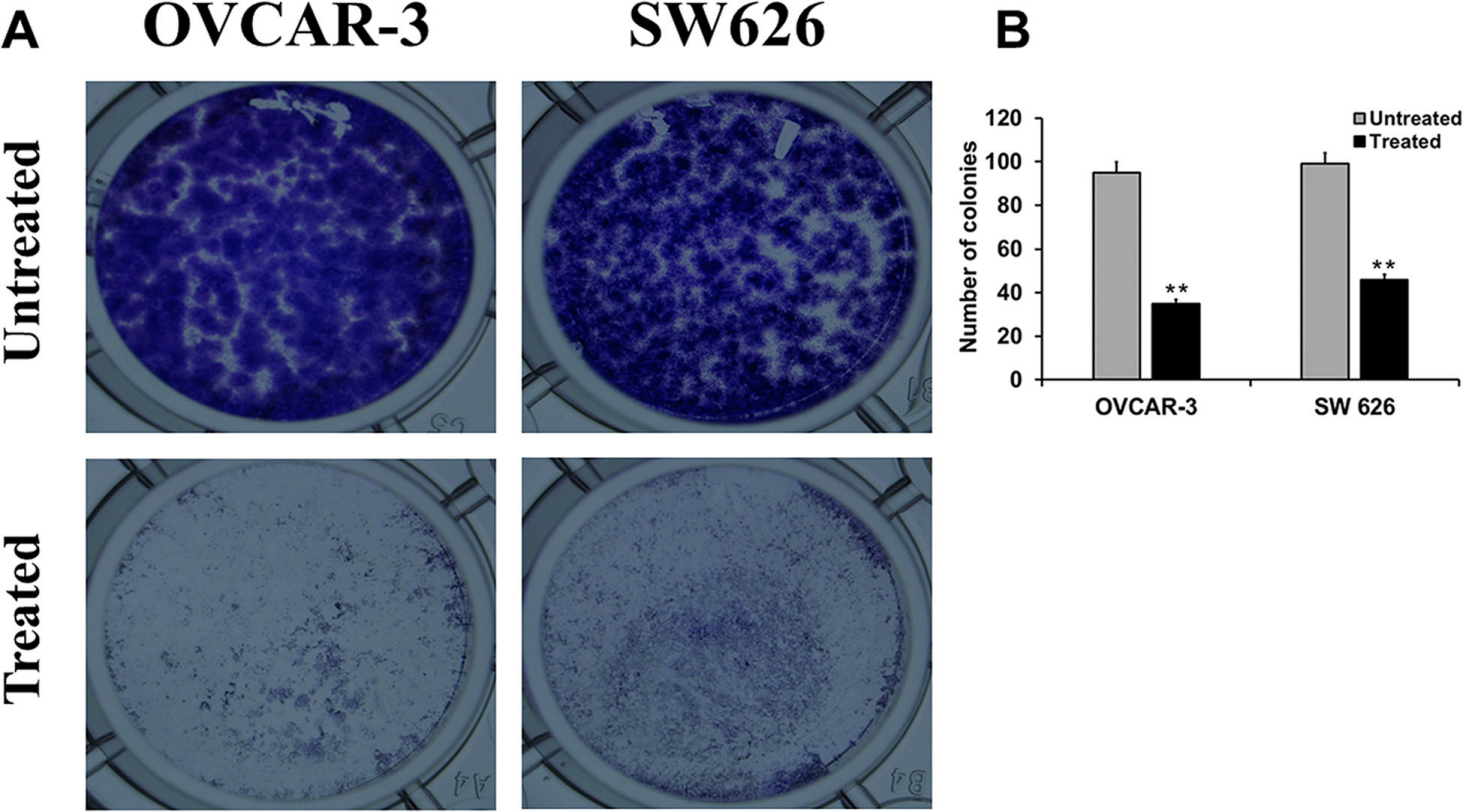
*B. coccineus* ethanol leaf extract suppresses colony formation in ovarian cancer cells. (**a**) OvCa cells were treated with *B. coccineus* extract and incubated for 10 days. Crystal violet 0.1% was used to stain the cells. A phase-contrasts light microscopy at 4X magnification was used to capture the images. (**b**) Quantitative data; the number of colonies containing over 50 cells were counted. Data presented as the Mean ± SEM, and level of significance was determined by students *t-* tests. The asterisks ** indicates *P* < 0.01. The experiments were repeated three times

To determine the effectiveness of cytotoxic agents, clonogenic or colony formation assay is the most applied method in which cell survival assay is based on the ability of a single cell to grow into a colony [13] To examine the effect of *B. coccineus* on colony formation, a clonogenic assay was carried out in OVCAR-3 and SW 626 cells. After treating and incubating for 10 days, the *B. coccineus* extract decreased the cell proliferation in all concentrations tested compared to untreated cells (Fig. 3a). In addition, we observed marked reduction in the cloning ability of OVCAR-3 (35%) compared to SW 626 (46%) cells (Fig. 3b). On other words, upon treatment with IC_50_ values, colony formation could be inhibited in OVCAR-3 and SW 626 OvCa cells,

### *B. coccineus* extract induces apoptosis in OvCa cells

**Fig. 4:**
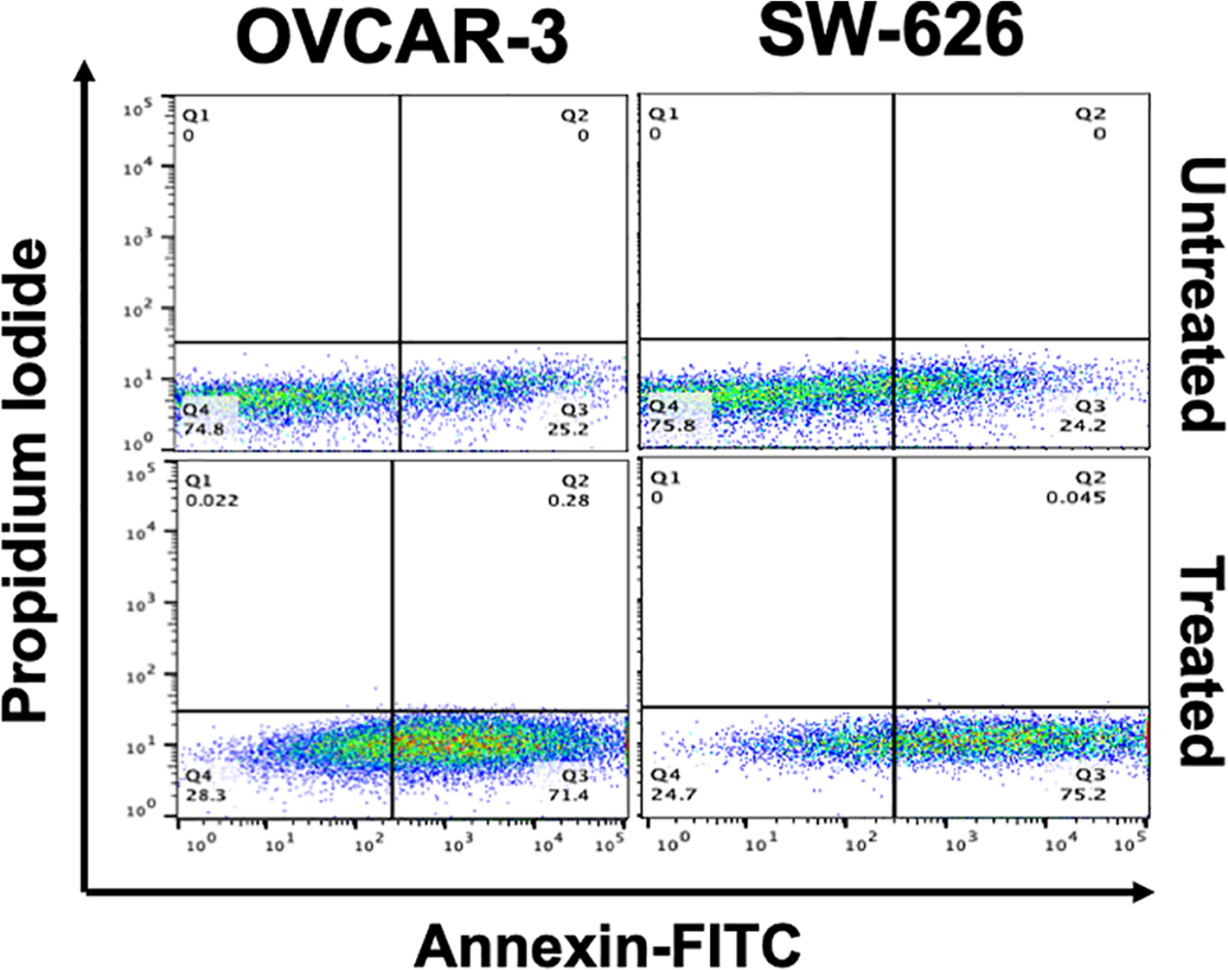
Effect of *B. coccineus* ethanol leaf extract on apoptosis of ovarian cancer cells. OvCa cells were treated with different dosage of *B. coccineus* extract (OVCAR-3: 446.5 μg/mL; and SW 626: 486.94 μg/mL) for 48 h, and apoptosis was assessed by staining with Annexin V-(conjugated with FITC)/ PI followed by flow cytometry. Quadrant Q1, Q2, Q3 and Q4 represents the percentages of necrotic (Annexin (−)/(PI (+), late (Annexin (+)/(PI (+), early (Annexin (+)/(PI (−) apoptotic, and viable (Annexin (−)/(PI (−) cells, respectively. DMSO was used in untreated cells. Upon treatment with extract, both cell lines showed higher number of apoptotic cells in early apoptotic phase compared to in untreated cells

To evaluate the cell death induced by *B. coccineus,* apoptosis assay was conducted in OVCAR-3 and SW 626 cell lines. The OvCa cells were treated with IC_50_ values for 48 h, stained with FITC**-**Annexin V/PI and then analyzed using the flow cytometer. As shown in Fig. 4, apoptotic cells were found more in the right lower (early apoptotic) quadrant (Q3) in the treated cells compared to untreated or control cells. The OVCAR-3 cells had 71.4% and 0.28% cells in early (Q3) and late (Q2) apoptotic phases respectively, while a total 25.2% cells were observed from both quadrants in untreated cells. Similarly, in SW 626 cells, upon a treatment, a total of 75.245% cells were found from the Q3 and Q2 quadrant, compared to 24.2% cells in the untreated group. Interestingly, minimal or no cells were observed in the necrotic phase (Q1) after the treatment. Moreover, the percentage of viable cells (Q4) was found to be high; 74.8 and 75.8% were in untreated OVCAR-3 and SW 626 cells, respectively. These findings further indicate that *B. coccineus* was able to induce apoptosis in OvCa cells.

### *B. coccineus* regulated gene expression at the mRNA level in OvCa cell lines

**Fig. 5:**
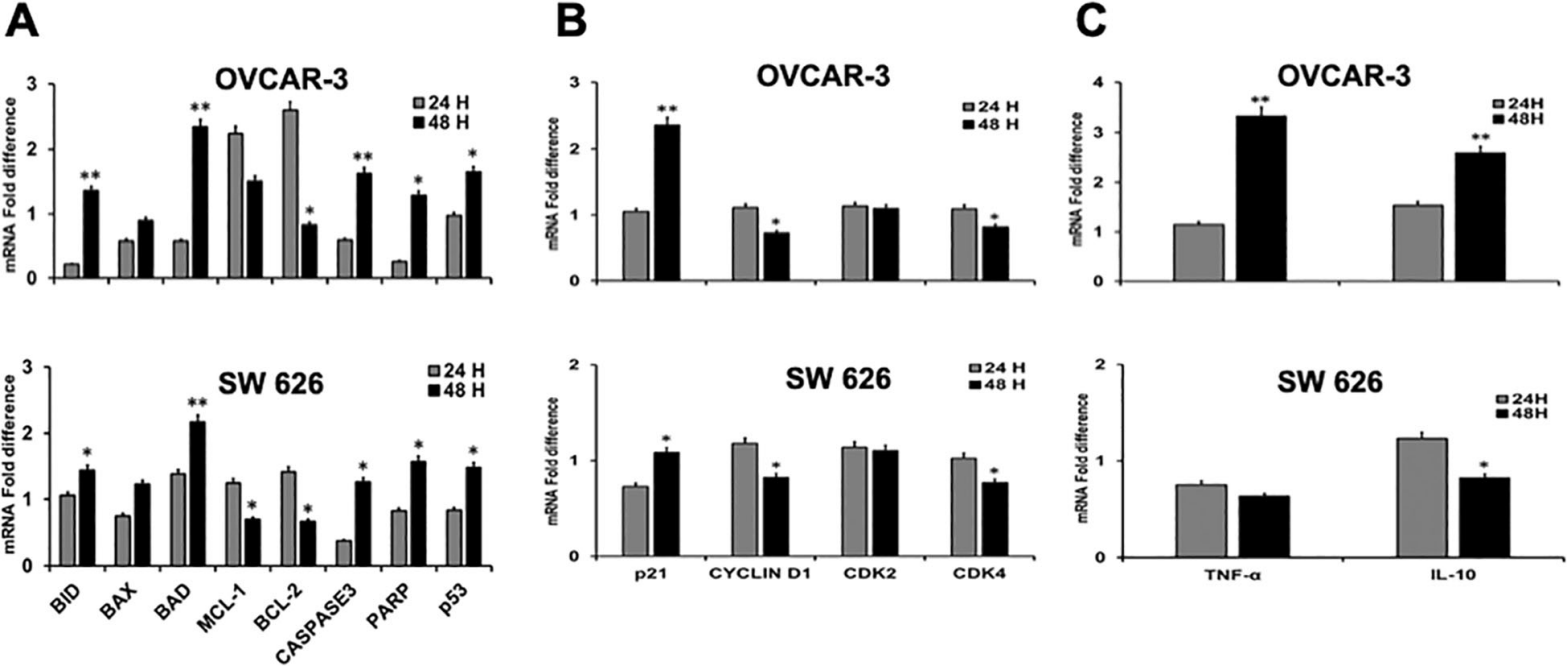
Validation of gene expression by RT-PCR for apoptosis, cell cycle and inflammatory markers. OVCAR-3 and SW 626 cells were treated with *B. coccineus* extract for 24 and 48 h, and mRNA fold change expression of (**a**) pro-(BID, BAX, BAD), anti- (MCL-1, BCL-2), apoptotic (Caspase-3, PARP), p53 (**b**) cell cycle regulators (p21, CYCLIND1, CDK2, CDK4), and (**c**) pro- inflammatory cytokines (TNFα, IL-10) markers, relative to control cells (DMSO used) were analyzed through RT-PCR. 18S, a house keeping gene was used for data normalization. Data presented as the Mean ± SEM, and level of significance was determined by students *t-* tests. The asterisks * and ** indicates *P* < 0.05 and *P* < 0.01, respectively. The experiments were repeated three times

The effect of *B. coccineus* ethanol extract on the expression of pro- and anti-apoptotic and tumor suppressor gene were investigated to determine if *B. coccineus* could induce apoptosis in OVCAR-3 and SW 626 cells. As shown in Fig. 5a, in OVCAR-3 and SW 626, upon treatment at 48 h, the expression of anti- apoptotic markers MCL-1 and BCL-2 were significantly down regulated to 1–2 folds in both cell lines (MCL-1 was non-significant in OVCAR-3), while were no effect on 24 h treatment. In turn, elevated the expression of pro- apoptotic markers (BID, BAX, and BAD) as shown in Fig. 5a. Further, we asked if pro-apoptosis would lead to execute apoptosis of OvCa cells. As Caspase-3 was used as an indicator for the cell death/apoptosis, we determined the mRNA levels of caspsase-3 in OvCa cells. The expression of Caspase-3 was significantly upregulated at 48 h indicating *B. coccineus* extract indeed promoting apoptosis in OvCa cells. In line of that, we examined the degradation of PARP as an indicator of apoptosis, treatment with extract in both cells enhanced proteolysis of PARP compared to 24 h.

Furthermore, we asked if *B. coccineus* extract inducing apoptosis in OvCa could lead to an increased level of tumor suppressor gene, p53. We investigated the mRNA transcript levels of p53, our data showed p53 was significantly enhanced at 48 h treatment compared to the 24 h. Although, there was no major difference in expression of p53 between OVCAR-3 and SW 626 cells. These results affirm that induction of apoptosis is linked with p53 expression in OvCa cells.

### *B. coccineus* modulates the cell cycle regulation at the mRNA level in OvCa cell lines

Small molecule inhibitors or natural products have been reported that simultaneous binding to both cyclin and CDK subunits can enforce cell cycle arrest in response to antiproliferation or cellular stress [14]. To investigate whether *B. coccineus* extract could arrest cell cycle in OVCAR-3 and SW 626 cells, we treated them with a known concentration of extract for 24 and 48 h time points. The cell cycle inhibitors p21 was found 1–2 folds upregulated at 48 h treated cells compared to 24 h (Fig. 5b). The increased expression of these inhibitors well supported the findings where p21 plays an important role in apoptosis by inducing pro- apoptotic genes and leads to cell cycle arrest [11]. In addition, to explore the changes in mRNA expression of cyclin D1/CDKs complexes that regulate the G1/S transition, we found significant inhibition in cyclin D1, however, CDK2 shows non-significant reduction in both cell lines. Furthermore, there was a significant decrease in CDK4 expression after 48 h of treatment with the extract (Fig. 5b). Altogether, our data demonstrate that *B. coccineus* extract blocks the cell cycle arrest and promote apoptosis via p53 dependent mechanism in OvCa cells.

The natural compounds inhibits the pro- inflammatory cytokines released by the activated immune cells [15, 16]. Therefore, we examined the effect of *B. coccineus* extract on Tumor necrosis factor-alpha (TNF-α) and IL-10, a multifunctional inflammatory cytokine, if they involved in OvCa cell survival, proliferation, and cell death. To do so, we treated OVCAR-3 and SW 626 cells with *B. coccineus* extract with their IC_50_ values (identified at 48 h) for two time-points 24 and 48 h. Surprisingly, in OVCAR-3, TNF-α and IL-10, both were upregulated several folds of mRNA expression at 48 h of treatment compared to 24 h (Fig. 5C). However, reduction in mRNA expression were found in SW 626 cells after 48 h. Although pro-inflammatory cytokines are not our focused area of study, while empirical findings support researchers to work on *B. coccineus* extract in immunomodulatory function.

### *B. coccineus* induces the regulation of pro- and anti-apoptotic protein expression in OvCa cells

**Fig. 6:**
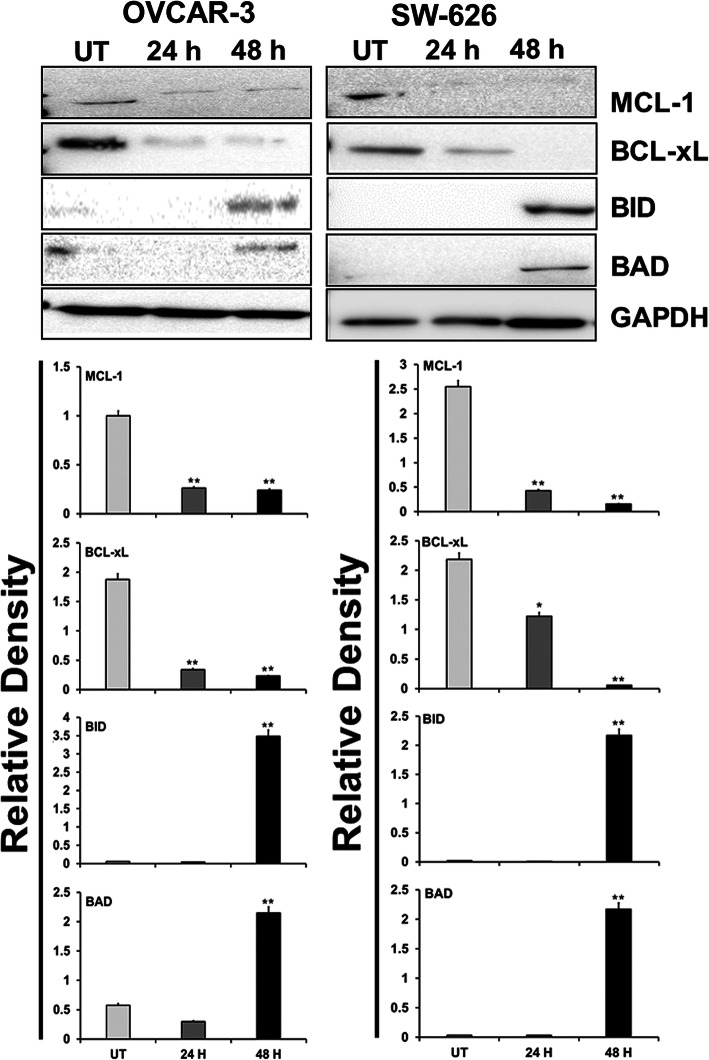
The expression of pro- and anti-apoptotic markers in ovarian cancer cells through western blots. OVCAR-3 and SW626 cells were treated with *B. coccineus* extract for 24 and 48 h, and the expression of apoptotic markers were analyzed through the western blots. The immunoblots of pro- (BID, BAD) and anti-apoptotic (MCL-1, BCL-X_L_) proteins are shown in upper panel, and the densitometry of the these proteins are shown in lower panel. DMSO was used in untreated (UT) cell. The immunoblots showed pro- apoptotic proteins were upregulated and anti-apoptotic were down regulated upon treatment at 48 h compared to 24 h and untreated cells. GAPDH, a house keeping genes was used as loading control to ensure equal loading in each blot. The asterisks ** indicates *P* < 0.01. The experiments were repeated three times

An accumulating evidence suggests that plant-based products promote apoptosis, inhibit metastasis, and suppress cancer growth, including OvCa [17, 18]. In view of these facts, we validated the involvement of *B. coccineus* in anti-proliferation and apoptosis-related genes in OvCa cells. OVCAR-3 and SW 626 cells were treated with the known concentration of *B. coccineus* extract for 24 and 48 h and analysed by western blots (Fig. 6). Our results indicate that the expression of pro- apoptotic genes (BAD and BID) were upregulated in OVCAR-3 cells treated for 48 h compared to 24 h and untreated cells; in turn, downregulates the expression of anti- apoptotic protein (BCL-xL and MCL-1). Interestingly, OVCAR-3 untreated cells showed, the least expression of BAD that could possibly be effect of DMSO. Likewise, in SW 626 cells, a marked increase in pro-apoptotic (BAD and BID) and decrease in anti- apoptotic protein (BCL-xL and MCL-1) were found at 48 h treated cells compared to 24 h or UT cells. Although, the extract was effective at 24 h treatment for BCL-xL but the expression was minimal in both cell lines. These results corroborate the finding of mRNA expression, where the *B. coccineus* leaf extract was found to be inducing apoptosis by inhibiting BCL-2 family gene and activating the pro-apoptotic genes. Thus, it confirms that *B. coccineus* extract promotes cell death in OvCa cells.

## Discussion

Among several cancers, epithelial ovarian cancer is the most lethal gynecologic malignancy. Identifying a targeted therapy for high grade serous (HGS) ovarian carcinoma with the drugs or other substances that attack cancer cells while no or little damage to normal cells is the utmost challenge from the decades. A tumor suppressor gene (p53) mutation was found to be associated with the development of various cancers including epithelial ovarian cancer as it regulates the cell progression and metastasis [18]. In addition, a recent report suggested a fallopian tube epithelium is the actual cause of this deadly disease than ovarian surface epithelium [19]. Thus, finding a compound or extract which can effectively enhance the p53 expression, suppress cell proliferation, and also has a killing effect on ovarian cancer cells is the main priority. *B. coccineus* plant extract preparation has been administered to patients traditionally in the management of various ailments including a tumors in West Africa [10]. The present study demonstrates that *B. coccineus* ethanol leaf extract targets multiple signaling pathways associated with cell proliferation, progression, and apoptosis in OVCAR-3 and SW 626 cell lines.

Several natural products have been found to exert an anti-cancer effect in OvCa, by inhibiting population growth and colony formation [20]. Previous studies have shown that by inducing apoptosis and cell cycle arrest through the down- regulation of Notch1, Notch3, Cdc25C, Akt, and Bcl-2 proteins inhibits OvCa cell proliferation [21]. Due to the anti-proliferative properties of *B. coccineus*, we hypothesized that the leaf extract could ameliorate the colony formation of a single cell in OvCa. Indeed, we reported a clonogenic assay that involves scoring and quantifying the colonies after treatment of *B. coccineus* in each cell line manually. Consistent with the published reports on cell viability and colony formation in breast and prostate cancer cell lines [10], which demonstrated decreased cell proliferation after *B. coccineus* treatment. We found that the treatment group had less colony compared to the vehicle control group in OVCAR-3 and SW-626 cells. In addition, to detect how the drug worked on cells we employed wound healing assay; *B. coccineus* plant extracts inhibited cells migration in both OvCa cell lines. Further, the cells were not migrated to the gap created by scratch when incubated with leaf extract, suggesting that both OvCa cell lines act on dose and time-dependent manner. This study is supported by Kan et al. 2018 [22], who showed that Sulforaphane treatment altered the scratch in OVCAR cells by suppressing cell migration and proliferation in a dose-dependent manner.

**Fig. 7:**
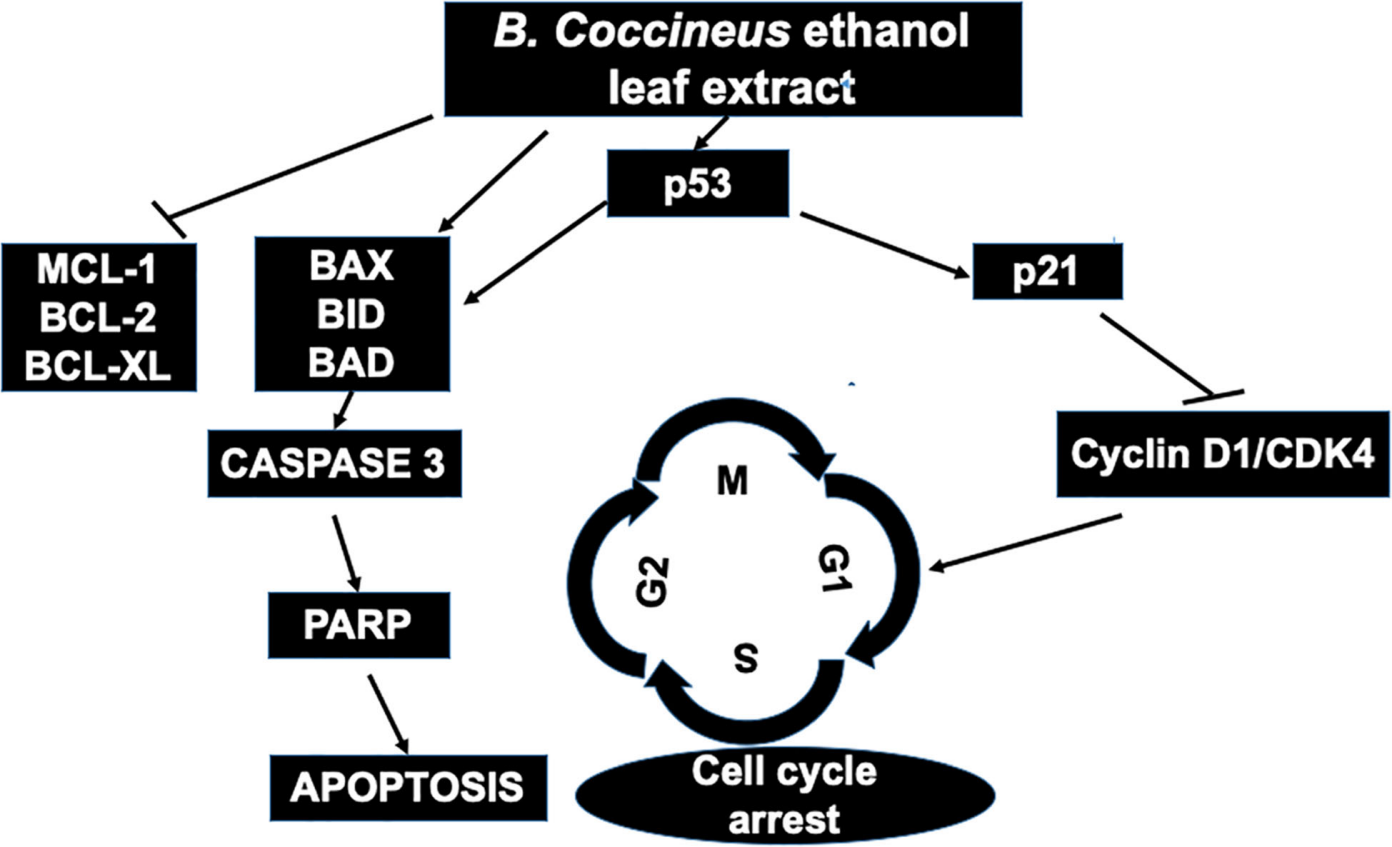
*B.coccineus* ethanol leaf extract induces cell cycle arrest and apoptosis via the p53 dependent pathway. As illustrated in model, p53 acts as transcription factor to regulate both apoptosis and cell cycle signalling cascade. The cell cycle progression is controlled by four phases: G1 or gap 1 phase , S or synthesis phase, G2 or gap2 phase and M or mitosis phase. Altogether, treating ovarian cancer cells with *B.coccineus* ethanol leaf extract could inhibit the cell proliferation, and increase an overall survival of patients

Apoptosis is important in the prevention of the proliferation of cancer cells. Extensive studies have shown that most cytotoxic agents with anti-proliferative activity regulates apoptosis [23]. Apoptosis is one of the important functions of p53 and it leads to disruption of tumor progression [23]. The p53, is important in cell cycle progression and apoptosis. The increased abundance of p53 by *B. coccineus* probably resulted in the up-regulation of pro-apoptotic markers (BAX, BID and BAD) and the down-regulation of anti-apoptotic markers (BCL-2, and MCL-1) as shown in this study. This finding corroborates with the fact that the up-regulation of p53 leads to the induction of apoptosis [11]. Furthermore, p53 regulates apoptosis through the extrinsic pathway, by up-regulating BID which inhibits anti-apoptotic proteins (BcL-2, BcL-XL and MCL-1) and intrinsic pathway by up-regulating BAX, shown in a model Fig. 7. We speculate that p53 also activates BAD which result in the release of BAX and its translocation to the mitochondria. BAX increases the permeability of the outer mitochondria membrane which leads to an efflux of cytochrome c. Cytochrome c then binds to Apaf-1 and initiates the initiator caspase 9 and this in turn initiates executioner caspases, such as caspases 3, 6, and 7. Caspase-3 cleaves PARP which leads to DNA fragmentation and then apoptosis [24]. To verify the speculation, we conducted qRT-PCR and evaluated its effect on apoptotic markers, caspase-3, and PARP. Consistent with another pro- apoptotic marker expression, the up-regulation of these further confirm the induction of apoptosis in treated cells.

Cell cycle inhibitors and cyclin-dependent kinases (CDKs) are the primary factors in the sequential transition of cells through G1, S, G2 and mitosis phases [14]. We showed that activation of p53 in OVCAR-3 and SW 626 cells treated with *B. coccineus,* arrest cell cycle by inducing the activation of p21 (Fig. 7). Herein, we observed p21 was up-regulated and cyclin D1 and CDK4 were significantly down-regulated in OVCAR-3 and SW 626 cell lines. p21, a CDK family inhibitor, inhibits the cell cycle at the G1 to S phase by binding to cyclin D/CDK4 and cyclin E/Cdk2 complex thereby preventing its progression [11]. The binding of p21 to the CDK4 and CDK2 results in their downregulation and this prevents the phosphorylation of Rb and leads to Rb forming a complex with a transcription factor, E2F1. This results in the inhibition of DNA replication and arrest of the cell cycle [25]. Studies have also shown that p21 can independently regulate pro-apoptotic proteins like BAX and anti-apoptotic proteins like BCL-2 without the expression of p53 [26]. The key point of this study is G1/S progression, one of the main checkpoints in the cell cycle regulation was found to be halted when OvCa cells were treated with *B. coccineus* leaf extracts.

We further investigated the association of *B. coccineus* leaf extracts with tumor-associated cytokines. Based on previous evidence, most of the immunosuppressants or stimulants are the chemically synthesized cytotoxic drugs, which possess the serious side effects and relapse [15]. Therefore, herbal medicines have always been important as therapeutic agents to modulate the immune system in the prevention of immune- related disease. TNF and IL-10, are multifunctional cytokines produced by the immune system, has a wide range of biological system including a pro- and anti-cancer effect [27]. TNF- α is the central and major mediators of the pro-inflammatory cytokine, our RT-PCR data suggests TNF-α was up-regulated in OVCAR-3 treated cells while were no significant effect found on SW 626 cells. It is worth mentioning that no reports are available on *B. coccineus* extract in the immunomodulatory function that needs to be further investigated. Studies have shown that TNF-α induces apoptosis through the extrinsic pathway by binding to TNFR which recruits the adaptor protein TNF receptor-associated death domain (TRADD) and Fas-associated death domain protein (FADD). These adopter proteins then transmit signals from TNFR to activate the caspase which leads to apoptosis [28]. TNF-α also induces apoptosis through the intrinsic pathway by cleaving of BCL-2 from BID which induces apoptosis via the intrinsic pathway [29]. Accumulation of p53 has been reported in some cells in which TNF-α has been shown to induce apoptosis, implicating p53 in TNF-α mediated apoptosis [30].

IL-10 which has both immunosuppressive and anti-angiogenesis effects has been shown in previous studies to stimulate TNF and other molecules of the immune system [31]. IL-10 was significantly up-regulated in OVCAR-3 treated cells, but not in SW 626 treated cells when compared to the control in these studies. IL-10 expression inhibits angiogenesis by down-regulating some molecules of the immune system like TNF-α which are needed for angiogenesis. This suggests that IL-10 did not play a major role in inducing apoptosis in SW 626 treated cells.

## Conclusion

It can be inferred in this study that *B. coccineus* ethanol leaf extract induces apoptosis probably by the expression of p53 and p21 genes and inhibits cyclinD1/CDK4 complex in OVCAR3 and SW626 cell lines. The p21 protein binds directly to cyclin-CDK4 complexes that drive forward the cell cycle and inhibits their kinase activity thereby causing cell cycle arrest to allow repair to take place.

The extract also inhibited ovarian cancer cell migration and colony formation. The *B. coccineus* also exhibited immune-modulatory properties by expressing TNF-α in the OvCa cell lines. All the results taken together suggest that *B. coccineus* induces apoptosis by upregulating p53 which results in cell cycle arrest and cell death.

## Methods

### Preparation of plant extract

Plant samples (wild type) were collected from the Southern part of Nigeria, and was authenticated by Prof. J. D. Olowokudejo, at the Dept. of Botany University of Lagos, Nigeria, with the Voucher Number 7491. The fresh leaves of *B. coccineus* were air dried and the powdered sample was extracted by maceration in ethanol. The filtrate was collected and evaporated to dryness using the evaporator at reduced pressure, to get the ethanol extract. Stock concentration of 20 mg/mL was prepared by dissolving 200 mg of ethanol leaf extract in 1 ml of DMSO and 9 mL of PBS. Stock solution was stored at − 20 °C.

### Materials

All chemicals used for this study were of analytical grade. All the primary and horseradish peroxidase-conjugated secondary antibodies were obtained from Cell Signalling Technology (Danvers, MA). Annexin V Apoptosis Detection Kit with PI was purchased from Bio-legend (San Diego, CA). The two-human epithelial, adherent ovarian cancer cell lines OVCAR3, and SW626 were obtained from ATCC (Manassas, VA).

### Cell cultures

The OvCa cell lines, OVCAR-3, and SW 626 were purchased from American Tissue Culture Collection (ATCC, Manassas, VA, USA). Following to the ATCC cell culture method with a small modification, OVCAR-3 cells were grown in RPMI media supplemented with 20% Fetal Bovine Serum (FBS) (Fisher scientific, PA, USA), 0.01 mg/ml bovine insulin, 1% of Non-essential amino acid solution and 1% mL of penicillin/streptomycin solution (Fisher Scientific, PA, USA). However, SW 626 cells were grown in L-15 media (Fisher Scientific, PA, USA), supplemented with 10% FBS, and penicillin/streptomycin solution. All cell cultures were maintained in a humidified incubator at 37 °C and 5% CO_2_.

### Cell proliferation assay

The MTT (3-(4,5-dimethylthiazol-2-yl)-2,5-diphenyltetrazolium bromide) (Fisher Scientific, PA, USA) assay was carried out to determine the viability of OVCAR-3 and SW 626 cells after *B. coccineus* ethanol leaf extract treatment. Both growing cells were trypsinized with 0.25% Trypsin-EDTA (Fisher Scientific, Pittsburgh PA), and seeded in 96-well plates at a cell density of 10,000 cells/well in triplicates. Cells were treated with different concentrations of *B. coccineus* ethanol leaf extract ranging from 0.020 mg/ml - 5 mg/ml each well per concentration, and incubated for 24, 48 and 72 h at 37 °C, and 5% CO_2_ incubator. DMSO (0.1%) used as a negative control. Next, 20 μL of MTT (5 mg/ml in PBS) reagent was added to each well and incubated for 2–3 h. The formazan crystals were solubilized in 100 μL of DMSO, and the optical density was measured at 570 nm using a microplate reader (Spectramax M5, Molecular Devices, Sunnyvale, CA) [12]. The optimal IC_50_ (half-maximum inhibitory concentration) values were calculated for both cell lines OVCAR-3 and SW 626.

### Live/dead cell staining

Twenty-five thousand OvCa cells were seeded in a 48-well plate overnight at 37 °C, and then treated with the plant extracts for 48 h. Following the manufacturer’s protocol (Thermo Fisher Scientific, Carlsbad, CA, USA), cells were washed and incubated with 2drops/mL of cell viability imaging probes for 15 min. The cells were then imaged using EVOS FL fluorescent microscope at 10X objective.

### Annexin V/PI apoptosis detection

Apoptosis assay detection kit with propidium iodide (PI) (Biolegend, San Diego, CA) was used to investigate if plant extract could induce cell apoptosis in OvCa cells. Both (OVCAR-3 and SW 626) cells were seeded (1 × 10^6^) in a six- well plate for 24 h and treated with an IC_50_ value of plant extract for 48 h. Further, cells were washed, harvested with 0.25% trypsin, and counted using the hemocytometer (Countess II FL, Life Technology, CA, USA). Subsequently, cells were washed twice with an ice -cold Annexin V binding buffer, and an equal number of cells (1 × 10^5^) were stained with Annexin V (conjugated with FITC) (5 μL) and PI (10 μL) in dark for 15 min at room temperature, according to manufacturer instructions. The Guava Flow cytometer (EMD Millipore, Brillarica, MA, USA) was used to acquire (50, 000 events/ sample) the frequency of apoptotic cells and analyzed using FlowJo 10.0.06 software.

### Wound healing assay

To investigate the effect of plant extract on OVCAR-3 and SW 626 cells migration, the wound- healing assay method was performed. Forty thousand (4 × 10^4^) cells/well were seeded in a 24 well plates, and placed in the 37 °C and 5% CO_2_ incubator for 24 h; when 90% cell confluency was achieved, a monolayer of the cells were scratched with a 10 μL plastic pipette tip to create a uniform wound. Subsequently, the cells were rinsed with PBS and incubated with *B. coccineus* extract (IC_50_ value) for 24, 48, and 72 h. To assess the migration from every scratch, a phase-contrast microscope (EVOS XL Core) (Thermo Fisher Scientific, Carlsbad, CA, USA), at 10X magnification was used.

### Clonogenic assay

Clonogenic or colony formation assay is based on the cells ability to form a colony from a single cell. The effect of the plant extract on OVCAR-3 and SW 626 colony formation was investigated. To determine, 1600 cells/well were seeded in 24 well plate and place in the incubator for 24 h. Cells were treated with extract and incubated at 37 °C for10 days. After washing with PBS, the cells were fixed with methanol for 20 min, stained with 0.1% crystal violet, and visualized using a phase-contrast light microscopy at 4X magnification, the formation of 50 cells and above were regarded as a colony.

### RNA isolation

Total RNA was isolated from OVCAR-3 and SW 626 cells treated with plant extract to determine its effect on apoptosis, cell cycle regulation and immunomodulation. The cells were treated for 24 h and 48 h, followed by washing with cold PBS, and lysed with lysis buffer. The binding, washing and elution of cells were performed as per manufacturer instructions using PureLink RNA Mini Kit (Thermo Fisher Scientific, USA). Next, RNA was suspended in nuclease-free water and quantified using the instrument Nanophotometer (Implen, Munich, Germany). cDNA was synthesized in a final reaction volume of 20 μL using 1.0 μg RNA and reverse transcription supermix for RT-qPCR according to the manufacturer protocol (Bio-Rad, Hercules, CA, USA). The primers sequences of all the genes used in this study were synthesized from the National Center for Biotechnology Information (NCBI) gene bank database. The primer sequences for MCL-1, BCL-2, BID, BAD, BAX, BAK, PARP, Cyclin D1, CDK2, p21, TNFα, IL-10, p53, and 18S are shown in Table 1. SYBR® Green PCR master mix reagents (Biorad, Hercules, CA, USA) were used for RT-PCR and gene expression was analyzed by CFX-manager software (CFX96 Real-Time System; Bio-Rad). All experiments were repeated three times.

**Table 1:** List of primer sequence

S. No.	Forward	Reverse
18S	GGCCCTGTAATTGGAATGAGTC	CCAAGATCCAACTACGAGCTT
MCL-1	5′-AAGAGGCTGGGATGGGTT TG-3′	5′-CAGCAGCACATTCCTGATGC-3′
BCL-2	5′-GATAACGGAGGCTGGGATGC-3’	5′- TCACTTGTGGCCCAGATAGG-3’
BID	5′-AGCACAGTGCGGATTCTGTC- 3’	5′-ACCGTTGTTGACCTCACAGT-3’
BAD	5′-CGAAGGGATGGGGGAGGA- 3’	5′-GGCGAGGAAGTCCCTTCTTA-3’
BAX	5′- AAACTGGTGCTCAAGGCCC-3’	5′-CTTCAGTGACTCGGCCAGG-3’
BAK	5′-TTTACCGCCATCAGCAACCT-3’	5′-ATAGGCATTCTCTGCCGTGG-3’
PARP	5′-GCTTCAGCCTCCTTGCTACA-3’	5′-TTCGCCACTTCATCCACTCC-3’
Cyclin D1	5′-TGCATCTACACCGACAACTC-3’	5′- TGGAGAGGAAGTGTTCAATG-3’
CDK2	5′-CGAGCTCCTGAAATCCTCCTG-3’	5′- GGCGAGTCACCATCTCAGCAA-3’
p21	5′- TGCCGAAGTCAGTTCCTTGT-3’	5′-GTTCTGACATGGCGCCTCC-3’
TNF-α	5′-ATGAGCACTGAAAGCATGATCC-3’	5′-GAGGGCTGATTAGAGAGAGGTC-3’
IL-10	5′-TCAAGGCGCATGTGAACT-3’	5′- GATGTCAAACTCACTCATGGCT-3’
p53	5′- TTTTCCCCTCCCATGTGCTC-3’	5′- TGGACGGTGGCTCTAGACTT-3’

### Western blot analysis

To determine the expression of pro- and anti- apoptotic proteins, OVCAR-3 and SW626 cells were treated with the plant extract for 24 h and 48 h time points. Cells were then washed with PBS, harvested, collected, and lysed with a RIPA buffer containing 1X protease and phosphatase inhibitors (Thermo Scientific, Rockford, IL). Following the standard protocol of protein isolation (Singh et al. 2019), the concentration of the total proteins was determined using BCA (bicinchoninic acid) protein assay kit (Thermo Scientific, Rockford, IL). The protein samples (30 μg) were denatured by heating in a Laemmli buffer and resolved on 12% SDS- PAGE. The separated proteins were transferred to PVDF membranes and incubated in blocking buffer (5% non-fat dry milk in TBS-T i.e. Tris-Buffered Saline containing 0.1% Tween-20) (Biorad, USA) for 1 h at room temperature. The membrane was then probed with primary antibodies (1:1000 dilution in 5% non-fat milk with TBS-T) against pro-apoptotic (BAD, BID), and anti-apoptotic (BCL-xL MCL-1) genes at 4 °C overnight followed by three wash with TBS-T. After washing, the membrane was incubated with horseradish peroxidase (HRP)-conjugated secondary antibodies (1:2000) dilution for 2 h at room temperature. The primary and secondary antibodies were purchased from (Cell Signaling Technology MA, USA). GAPDH was used to ensure equal loading. The chemiluminescent reagent (Thermo Fisher Scientific, Rockford, IL) was added to the membrane to detect and visualize the proteins using Image Quant LAS4000 (GE Healthcare- Biosciences, Pittsburgh, PA) [3].

### Statistical analysis

Statistical analysis was done by using a one-way analysis of variance (ANOVA) and unpaired *t-*tests. Results are expressed as standard errors of means (±SEM). *P* values less than 0.05 were considered statistically significant.

## Supplementary information

**Additional file 1: Figure S1**. Effect of *B. coccineus* ethanol leaf extract on cell viability of ovarian cancer cells. OvCa cells (OVCAR-3 and SW 626) were grown and treated with different dosage of *B. coccineus* extract (0.019, 0.039, 0.078, 0.156, 0.312, 0.625, 1.25 μg) for 24, 48 and 72 h. DMSO was used as vehicle control. Thereafter, 5 mg/mL MTT (3-(4,5-dimethylthiazol-2-yl)-2,5-diphenyltetrazolium bromide) was applied to determine the viability of cells. The formazan crystals were solubilized in 100 μL of DMSO. The optical density was measured at 570 nm using a micro-plate reader (Spectramax M5, Molecular devices, Sunnyvale, CA). The optimal IC_50_ (half-maximum inhibitory concentration) values were calculated for both cell lines OVCAR-3 and SW 626. The IC_50_ values of extract were found to be 446.5 μg/mL and 486.94 μg/mL for OVCAR-3 and SW 626 cells, respectively at 48 h timepoint. However, at 72 h, it was 443.1 and 469 μg/mL for OVCAR-3 and SW 626 cells, respectively.

## Data Availability

All datasets generated during this study are included in this article.

## Abbreviations

OvCa: Ovarian cancer cell
GAPDH: Glyceraldehyde 3-phosphate dehydrogenase
HRP: Horseradish peroxidase
FBS: Fetal Bovine Serum
MTT: 3-(4,5-dimethylthiazol-2-yl)-2,5-diphenyltetrazolium bromide
NCBI: National Center for Biotechnology Information
TNF-α: Tumor necrosis factor alpha
