# Supplementary figures and images for: Anti-proliferative activities of Byrsocarpus coccineus Schum. and Thonn. (Connaraceae) using ovarian cancer cell lines
Source: J Ovarian Res. 2020 Jul 21;13:83. doi: 10.1186/s13048-020-00679-8 (PMC7374866; doi:10.1186/s13048-020-00679-8)

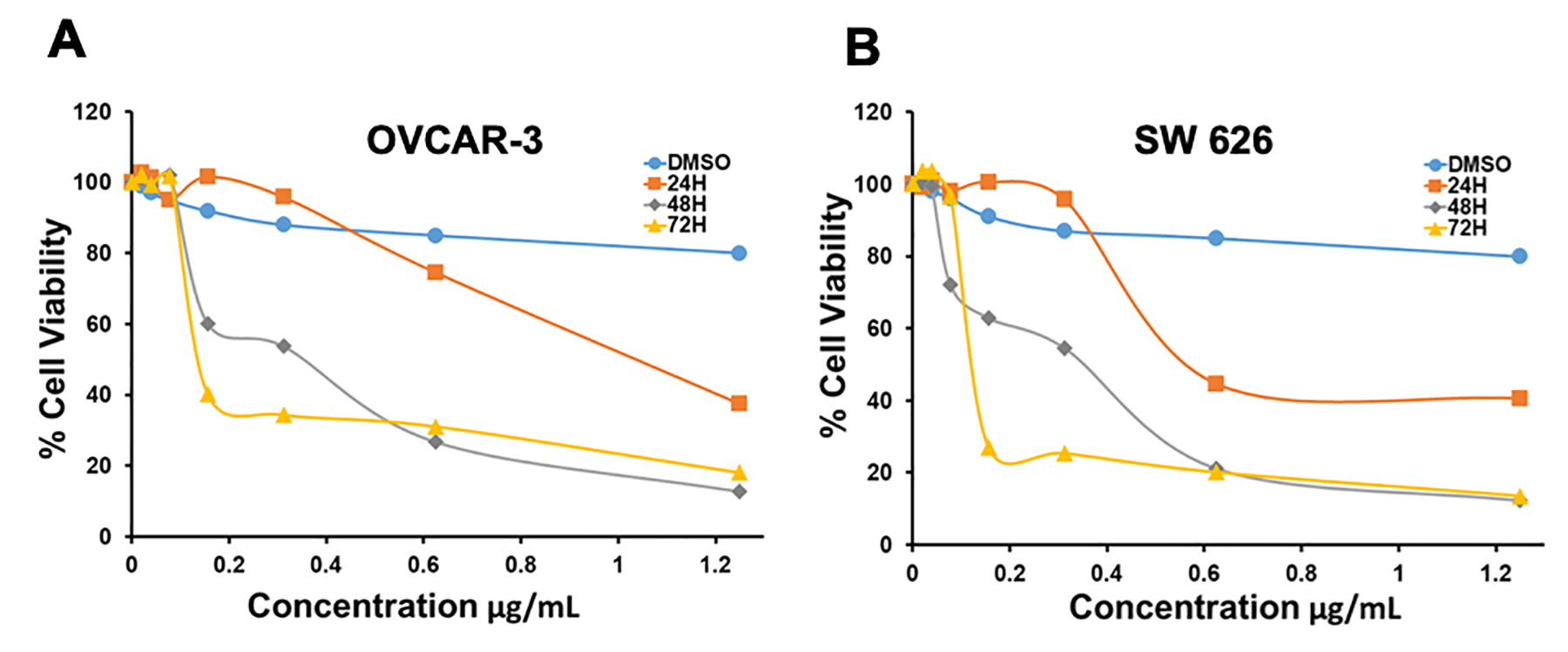

Supplement: Supplementary file 1 — Additional file 1: Figure S1. Effect of B. coccineus ethanol leaf extract on cell viability of ovarian cancer cells. OvCa cells (OVCAR-3 and SW 626) were grown and treated with different dosage of B. coccineus extract (0.019, 0.039, 0.078, 0.156, 0.312, 0.625, 1.25 μg) for 24, 48 and 72 h. DMSO was used as vehicle control. Thereafter, 5 mg/mL MTT (3-(4,5-dimethylthiazol-2-yl)-2,5-diphenyltetrazolium bromide) was applied to determine the viability of cells. The formazan crystals were solubilized in 100 μL of DMSO. The optical density was measured at 570 nm using a micro-plate reader (Spectramax M5, Molecular devices, Sunnyvale, CA). The optimal IC50 (half-maximum inhibitory concentration) values were calculated for both cell lines OVCAR-3 and SW 626. The IC50 values of extract were found to be 446.5 μg/mL and 486.94 μg/mL for OVCAR-3 and SW 626 cells, respectively at 48 h timepoint. However, at 72 h, it was 443.1 and 469 μg/mL for OVCAR-3 and SW 626 cells, respectively. [file 13048_2020_679_MOESM1_ESM.tiff]
